# Price Decision-Making in Dual-Channel Healthcare Services Supply Chain Considering the Channel Acceptance, Price Ceiling, and Public Welfare

**DOI:** 10.3390/ijerph192013028

**Published:** 2022-10-11

**Authors:** Yanbo Ma, Zheng Li, Kaiyue Liu, Zhengmin Liu

**Affiliations:** School of Management Science and Engineering, Shandong University of Finance and Economics, Jinan 250014, China

**Keywords:** healthcare services, dual-channel, price decision-making, price ceiling, Stackelberg game, public welfare

## Abstract

Given that an increasing number of online healthcare channels play an essential role as a supply method in the healthcare service supply chain (HSSC), this paper studies the price decision-making problem for a dual-channel HSSC considering the channel acceptance, price ceiling, and public welfare. In this HSSC, a healthcare institution establishes both a traditional offline channel and an online channel to provide healthcare services for some health conditions. Considering the public welfare of healthcare institutions, we employ a sum formula of economic revenue and patient surplus to describe the total revenue of both healthcare service channels. Based on the Stackelberg game, we develop a decentralized supply chain model to maximize supply chain members’ revenue. By employing the Karush–Kuhn–Tucker optimality condition, we derive an analytical expression for the optimal service price, which includes the functions of the public welfare coefficient and channel acceptance. Finally, we conduct extensive numerical analyses under various system parameters to verify the optimal price decision-making strategies. Our analytical results indicate that: (1) the healthcare service price is closely related to the patients’ channel acceptance, the public welfare coefficient, and the government price ceiling policy; (2) the public welfare coefficient strongly influences the service price and total revenue, and its increase can decrease the economic revenue of the HSSC; (3) the acceptance of online channels is an essential factor that should be carefully considered in the construction of a dual-channel HSSC. Improving patient acceptance of online channels is conducive to developing and improving a sustainable dual-channel HSSC.

## 1. Introduction

Recently, unprecedented and significant changes have taken place in online healthcare channels due to the development of the Internet. In particular, it has offered patients more choices in terms of healthcare services and alleviated their difficulties in seeking healthcare services due to the uneven distribution of healthcare resources. It also provides new ideas for the sustainable development of healthcare service supply channels. A market analysis report by Deloitte Consulting shows that people’s attitudes toward Internet diagnosis and treatment have undergone positive changes in the post-epidemic era, accelerating patients’ tendency to use Internet healthcare services [1]. Notably, due to the COVID-19 epidemic, an increasing number of online healthcare channels, e.g., online appointment and consultation platforms, are being gradually integrated into the healthcare market and play a significant role as suppliers in the healthcare services supply chain (HSSC). Furthermore, stimulated by the state’s support of the “Internet+” healthcare policy, more and more hospitals in China, such as the Affiliated Hospital of Sun Yat-sen University, Peking Union Healthcare College Hospital, and Shandong Provincial Hospital, have established online healthcare channels to implement the dual-channel healthcare services strategy and try to achieve the sustainable development of healthcare services supply.

As a new healthcare service supplier, the online channel usually consists of various services, including pharmaceuticals, catering, and healthcare consumables [2]. It is mainly constituted by healthcare institutions and patients and is under the supervision of the government [3]. Moreover, the online channel has gradually altered the mode by which patients access healthcare services [4,5,6]. Simultaneously, new challenges have arisen in the operation of the HSSC and its sustainable development, such as the market demand competition between the online and offline channels, the price decision-making mechanism of the dual-channel strategy, and the allocation of public welfare between the online channel and the offline channel. Therefore, a reasonable price decision-making strategy urgently needs to be proposed to coordinate the competition between online channels and offline channels, guaranteeing that patients can obtain more public welfare in the online channel. Moreover, a good service price decision-making strategy is also conducive to developing a dual-channel HSSC.

In this paper, we employ game theory to explore the optimal price decision while considering the combined effect of the patients’ channel acceptance, the public welfare, and the government’s price ceiling policy. Motivated by existing related research, we address the price decision-making problem for a real HSSC, where both the online and offline channel provide healthcare services for specific patients’ demands. Specifically, we develop an analytical supply chain model to examine the effects of patients’ channel acceptance, public welfare, and the government’s price ceiling policy on supply chain members’ revenue. The offline channel is the Stackelberg price decision leader, and the online channel is the follower. Our findings indicate that the public welfare coefficient and patients’ channel acceptance significantly affect the optimal service price strategy. Considering the overall revenue of the dual-channel HSSC and the maximization of patient surplus, the optimal price decision-making strategy is proposed considering the government’s price ceiling policy. This strategy provides theoretical support and a price decision-making basis for the improvement and development of the dual-channel HSSC. The novel contributions of this paper are listed as follows:

▴ Firstly, we provide an analytically tractable framework for the dual-channel HSSC, where the combined effect of the patients’ channel acceptance, the public welfare, and the government’s price ceiling policy on the price decision-making for the dual-channel system are considered. In the framework, we employ a sum formula of economic revenue and patient surplus to describe the total revenue of the HSSC. To the best of our knowledge, this work is one of the earliest attempts to address the issue of price decision-making strategies for the dual-channel HSSC.
▴ Based on the developed framework, we formulate the healthcare service price decision-making strategy for the studied dual-channel HSSC, intending to maximize the sum of economic revenue and patient surplus while considering the price ceiling. Moreover, via employing the Karush–Kuhn–Tucker(KKT) optimality conditions, the optimal healthcare service price decision-making strategy is derived in a closed form. It is noted that this price decision-making strategy reveals the inherent trade-off between the revenue of the healthcare services supply chain and its public social welfare.
▴ We conduct an in-depth numerical analysis to provide insights into the influence of the patients’ channel acceptance, public welfare, and the government’s price ceiling policy on price decision-making for both the online and offline channels. Furthermore, the results highlight the patient surplus achieved by the dual channels. Numerical results verify the effectiveness of the proposed price decision-making strategy by comparing it with a benchmark single-channel HSSC.

The paper is organized as follows. Section 2 reviews and summarizes the relevant literature. Section 3 presents the system model for the dual-channel HSSC scenario and the formulation of the utility functions. In Section 3, we formulate the single-channel healthcare services model and analyze the effect of the public welfare coefficient as a benchmark of the dual-channel model. An analytical solution is derived by invoking the Stackelberg game and optimization theory to address the optimal price strategy proposal in Section 4. The numerical results and the rationality of the optimal price strategy are presented in Section 5. Finally, in Section 6, we conclude the paper and propose the future research direction.

## 2. Literature Review

The issue of price decision-making in the healthcare services supply chain has been extensively researched in recent years. Firstly, healthcare institutions have a limited area of action, leading to large hospitals’ apparent siphoning effect. The mismatch between the service cost and service price compensation of healthcare institutions leads to considerable differences in price decision-making [7,8,9]. Many scholars have proposed different price strategies and formulated models according to national conditions. Duan J.L et al. [10] established a game price decision-making model considering patients’ elasticity of demand and quality preference, and the study showed that the equilibrium price was correlated with the coefficient of patients’ quality preference. Dan B et al. [11,12] established a drug price decision-making model for the medicine supply chain composed of drug suppliers and healthcare institutions under different channels, and the influence of drug price ceiling policies and the public welfare of healthcare institutions on the changes in drug prices was investigated. Grennan [13] proposed a method of service price decision-making based on the relationship between doctor–patient supply and demand, as well as a competitive price decision method based on reference to the service prices of other healthcare institutions. Kouveils et al. [14] established a drug supply chain problem for a multi-drug benefit manager based on the MNL model and studied the competition phenomenon, and they obtained the optimal strategy by solving the model analytically. Lillrank et al. [15] established a model to analyze patients’ selection of healthcare service quality based on the different price control strategies in hospitals. Sinha et al. [16] focused on the quality and cost of healthcare services and formulated a healthcare insurance strategy analyzed based on game theory. Gao L.Y et al. [17] established a healthcare services supply chain network consisting of the government, health insurance funds, hospitals, and patients. This paper studied the impact of reference price and health insurance reimbursement strategy dialogues on patients’ choices and healthcare service supply networks.

Research conducted using empirical and modeling methods on dual-channel supply chains in non-service industries is also well established. Khouja et al. [18] studied the problem of channel selection and price decision-making in the dual-channel model of traditional retail industries, and the impact of cost changes on channel selection and channel price was obtained. Quon et al. [19] analyzed the phenomenon of consumers purchasing medicine through the online channel. They concluded that the low price is the reason for the transfer of the common channels for consumers to buy drugs. Desai et al. [20] indicated that consumers will ignore the online consultation link, which is related to the patients’ acceptance and cognition of healthcare services through online channels. P. Mala et al. [21] demonstrated a mathematical model in the form of a constrained non-linear program and solved it by employing the Lagrangian method. Ata Allah Taleizadeh et al. [22] focused on the dual-channel supply chain model, where a manufacturer is regulated by the cap and trade system. This study raises the importance of inter-channel cooperation. Modak et al. [23] studied the impact of social public responsibility factors on consumers’ choices in the current global business environment via modeling methods. Yue B.C [24] constructed a multi-channel supply chain system from the perspective of channel rights and customers’ channel preferences and established a multi-channel linear demand function based on the channel preference coefficient. This paper studied the optimal channel structure selection problem for different decision-making aspects.

A few scholars have also addressed patient behavior in the supply chain of healthcare services and examined the dual-channel structure of healthcare services. Wang X.L et al. [25] constructed a dual-channel healthcare delivery system by considering patients’ delay sensitivity and transportation costs, and the study concluded that the dual-channel healthcare system is more flexible and sustainable than the traditional outpatient and gatekeeper systems. Through the empirical analysis method, Fittler et al. [26] analyzed consumers’ choices of drug purchase channels and concluded that the online and offline channel medicine purchase differences of patients will affect the patient choice. This paper also suggests that the online channel should improve the healthcare consultation service.

The existing literature has laid a solid foundation for price decision-making in the single-channel healthcare service structure, price decision-making in the dual-channel supply chain structure, and the influence factors for the dual-channel supply chain of non-service products. However, considering that healthcare services are a particular type of trust product, the demand for the HSSC is heavily influenced by the choice behavior of patients and the unique nature of the healthcare service. Therefore, these works on the dual-channel supply chain cannot be directly extended to the healthcare services supply chain. In this paper, we address the issue of price decision-making while considering the patients’ behavior and the specific nature of healthcare services.

## 3. Dual-Channel HSSC and Assumptions

**Figure 1:**
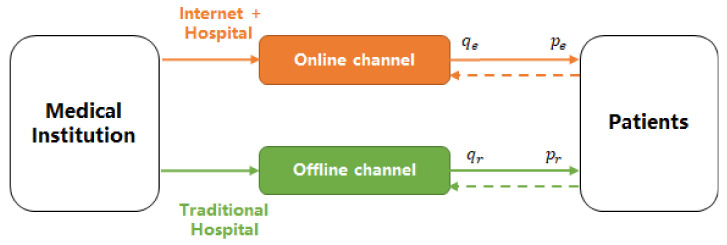
Dual-channel HSSC structure.

We consider a dual-channel HSSC scenario, as illustrated in Figure 1. A healthcare institution establishes both an offline and online channel to provide healthcare services for some given health conditions, whereas an online channel healthcare service is provided via the Internet. It is supposed that the doctors who provide given healthcare services on both the offline and online channel are the same, so as to ensure that patients receive equally effective healthcare services online and offline. We assume that patients must seek healthcare when they are affected by the given health condition, and the patients’ population is standardized to 1. In this way, patients will have alternatives when seeking healthcare services. One option is seeking offline healthcare treatment, i.e., going to the healthcare institution, and the other is making an online appointment and conducting a consultation through the online channel. We denote the healthcare treatment price paid on the offline channel as $p_{r}$ and the price paid on the online channel as $p_{e}$. Without loss of generality, we assume $p_{e}\leq p_{r}$. We introduce μ to denote the self-utility of the patient when he/she is healthy. In addition, the disease severity condition of the patient is denoted by δ, which is supposed to obey a [0,1) distribution. In the extreme, δ is 0 and corresponds to the case wherein the patient is in a healthy state, while δ is 1, corresponding to the case wherein the patient’s utility is 0. For details, readers are referred to [27]. Thus, δμ can be used to represent the lost utility of patients after an illness, and it is the maximum utility that the patient can obtain from receiving healthcare services. To simplify the results, we assume that the patient’s healthy utility is μ=1, and then, the lost utility of the patient can be simplified to δ. We also assume that the patient’s maximum recovery degree when receiving services can reach 100%.

Considering that the offline channel has been the main, traditional means of healthcare treatment for a long time, it is assumed in this paper that the patients’ acceptance of the offline channel is 1 and that of the online channel is θ, and θ obeys a uniform distribution of (0,1) [28]. This distinction is due to the fact that patients’ willingness to adopt the online channel varies from person to person [29]. The recovery degree of receiving healthcare services through the online channel is presumed to be ρ, and 0<ρ<1, due to the differences in doctor–patient information exchange and healthcare equipment between the online and offline channel. According to the above assumptions, the utility function obtained by a patient after receiving healthcare services through offline channels is given as

$$U_{r}=\delta -p_{r}.$$

Moreover, the utility function obtained after receiving healthcare services through the online channel is

$$U_{e}=\theta \rho \delta -p_{e}.$$

We assume that patients are rational in this paper. Thus, each patient can choose one of the two channels for healthcare services according to the utility maximization principle, i.e., $U_{max}=max\left\{U_{e},U_{r}\right\}$, where $U_{max}$ denotes the maximum utility that a patient can obtain.

**Figure 2:**
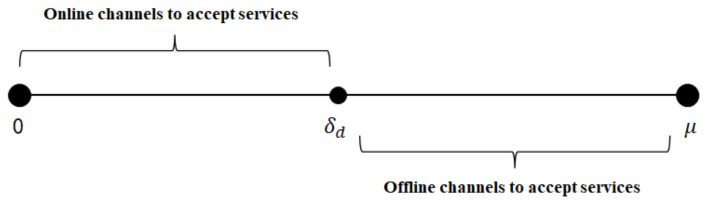
Patient channel selection under dual-channel strategy according to μ.

Specifically, as shown in Figure 2, when $0<\delta^{*}<\delta_{d}$, patients are more receptive to online channels, and when $\delta_{d}<\delta^{*}<1$, patients are more receptive to offline channels. When $\delta_{d}=\left(p_{r}-p_{e}\right)/\left(1-\theta \rho \right)$, patients can choose either the online channel or offline channel for healthcare services. Thus, the patients’ healthcare services demand for the online and offline channel can be described as:

$$q_{e}=\delta_{d}-0=\frac{p_{r}-p_{e}}{1-\theta \rho },$$

$$q_{r}=1-\delta_{d}=1-\frac{p_{r}-p_{e}}{1-\theta \rho },$$

where $q_{e}>0,q_{r}>0$, i.e., patients’ demand for online and offline channels exists simultaneously. We can deduce that this requires $p_{e}+1-\theta \rho >p_{r}>p_{e}$ to be satisfied. In the healthcare industry, to control the price of services and avoid the excessive marketization of healthcare services, the government usually adopts a price ceiling policy for healthcare services. In this paper, we assume that the price ceiling of healthcare services is $p_{0}$, and $p_{e}\leq p_{r}\leq p_{0}\leq 1$. Commonly, due to the social welfare characteristics of healthcare services, there will be a situation wherein the revenue of healthcare institutions does not cover the expenses. Then, the government will use the financial subsidy policy to compensate, which means that the negative economic revenue will not affect the regular operation of the healthcare institution. This paper refers to the markup price decision-making method of healthcare institutions. It assumes that the healthcare service price of the online and offline channels consists of two parts: the unit cost and unit revenue. Mathematically, $p_{e}=a_{e}+c_{e},p_{r}=a_{r}+c_{r}$, where $a_{e}$ and $a_{r}$ represent the unit revenue of the online channel and the offline channel, respectively, and $c_{e}$ and $c_{r}$ represent the unit service costs of the online channel and the offline channel, respectively, where $c_{r}>c_{e}$. $a_{e}$ and $a_{r}$ are decision variables in the model.

Therefore, under the dual-channel HSSC strategy, the profit function of each channel can be expressed as:

(1) $$\begin{array}{cc} & \pi_{e}=a_{e}\cdot q_{e}=a_{e}\cdot \frac{p_{r}-p_{e}}{1-\theta \rho }, \end{array}$$

(2) $$\begin{array}{cc} & \pi_{r}=a_{r}\cdot q_{r}=a_{r}\cdot \left(1-\frac{p_{r}-p_{e}}{1-\theta \rho }\right), \end{array}$$

where $\pi_{e}$ and $\pi_{r}$ denote the economic revenue of the online channel and the offline channel, respectively. We assume that the healthcare institution is commonly a public welfare organization whose key feature is public welfare. Referring to the research [30] and synthesizing previous studies on consumer surplus [31,32], the patient surplus of the HSSC can be expressed as:

(3) $$\begin{array}{cc} & CS_{e}=\int_{0}^{\delta_{d}}\left(\delta -p_{e}\right)d\delta , \end{array}$$

(4) $$\begin{array}{cc} & CS_{r}=\int_{\delta_{d}}^{1}\left(\delta -p_{r}\right)d\delta , \end{array}$$

where $CS_{e}$ and $CS_{r}$ denote the patient surplus generated by receiving healthcare services through the online and offline channel separately. Let β indicate the public welfare coefficient of the healthcare services industry under government supervision, where β∈[0,1]. The larger the β value, the higher the public welfare of healthcare services is.

Then, we can calculate the channels’ total revenue $v_{e}$ and $v_{r}$:

(5) $$\begin{array}{cc} & v_{e}=\pi_{e}+\beta \cdot CS_{e}=a_{e}\cdot \frac{p_{r}-p_{e}}{1-\theta \rho }+\beta \cdot \int_{0}^{\delta_{d}}\left(\delta -p_{e}\right)d\delta , \end{array}$$

(6) $$\begin{array}{cc} & v_{r}=\pi_{r}+\beta \cdot CS_{r}=a_{r}\cdot \left(1-\frac{p_{r}-p_{e}}{1-\theta \rho }\right)+\beta \cdot \int_{\delta_{d}}^{1}\left(\delta -p_{r}\right)d\delta . \end{array}$$

The key parameters used in this paper are summarized in Table 1.

## 4. Model Analysis

This section considers the single-channel strategy as a benchmark scenario in which there is no channel choice behavior. Then, we investigate a dual-channel strategy where the online and offline channels exist simultaneously. Next, we analyze the optimal service price decision-making strategy under different channel strategies and explore the impact of the patients’ acceptance of the online channel, price ceiling, and public welfare coefficient on the optimal service price strategy. The methods used in this paper are shown in Table 2.

### 4.1. Benchmark Scenario: Single-Channel Strategy

We first formulate a single-channel HSSC model where patients can only choose the offline channel for healthcare services. We denote the price of healthcare services as $p_{s}$, $p_{s}=a_{s}+c_{s}$, where $a_{s}$ and $c_{s}$ denote the unit revenue and the service cost, respectively. In a single-channel HSSC, the utility obtained by a patient after receiving healthcare services is given as

$$U_{s}=\delta_{s}\cdot \mu -p_{s}.$$

**Figure 3:**
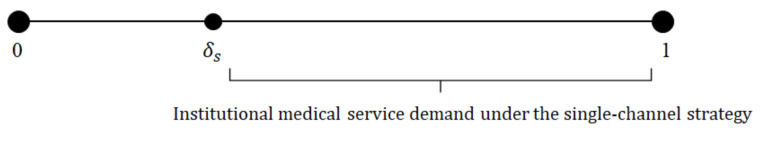
Institutional healthcare service demand in single-channel strategy.

We assume that patients can choose to receive healthcare services or not according to the degree of their illness. Specifically, as shown in Figure 3, when $0<\delta^{*}<\delta_{s}$, patients will not receive healthcare services; when $\delta_{s}<\delta^{*}<1$, patients choose to receive healthcare services; when $\delta_{s}=p_{s}$, patients receive the same utility as those who do not receive medical services. Thus, we can easily obtain the patients’ healthcare service demand: $q_{s}=1-\delta_{s}=1-p_{s}$ [27].

Thus, we can express the healthcare institution’s economic revenue and total revenue, respectively, as:

(7) $$\begin{array}{cc} & \pi_{s}=a_{s}\cdot q_{s}=a_{s}\cdot \left(1-p_{s}\right)=a_{s}\cdot \left(1-a_{s}-c_{s}\right), \end{array}$$

(8) $$\begin{array}{cc} & v_{s}=a_{s}\cdot q_{s}+\beta \cdot \int_{\delta_{s}}^{1}\left(\delta -p_{s}\right)ds. \end{array}$$

From Equation (7), we can obtain $\frac{d^{2}\;\pi_{s}}{d\;a_{s}^{2}}=-2$, then it is obvious that $\pi_{s}$ is strictly concave in $a_{s}$ [33]. Moreover, by setting $\frac{d\;\pi_{s}}{d\;a_{s}}=1-2a_{s}-c_{s}=0$, we can obtain the unit revenue that maximizes the economic benefit of the single-channel as $a_{s}^{{}^{\prime }}=\frac{1-c_{s}}{2}$; then, we can obtain $p_{s}^{{}^{\prime }}=1+a_{s}^{{}^{\prime }}=\frac{1+c_{s}}{2}$. Healthcare institutions aim to maximize the total revenue, which consists of economic revenue and public welfare. Then, we introduce the public welfare coefficient to obtain the total revenue, and we formulate the model. The single-channel HSSC price decision-making model can be expressed as

(9) $$\begin{array}{ccc} & \max & v_{s}=a_{s}\cdot q_{s}+\beta \cdot \int_{\delta_{s}}^{1}\left(\delta -p_{s}\right)d\delta ,\\ & s.t. & p_{s}\leq p_{0},\\ & & a_{s}>0, \end{array}$$

where the first term in $v_{s}$ denotes the economic revenue for offering healthcare services and the second term denotes the revenue of public welfare. We can observe that the total revenue of the single channel depends on the optimal service price and public welfare coefficient. Constraints specify that the optimal service price should not exceed the price ceiling, and the unit revenue is non-negative.

The objective function of Equation (9) can be expand as $v_{s}=a_{s}\cdot \left(1-a_{s}-c_{s}\right)+\beta \cdot \left(\left(\frac{1}{2}-\left(a_{s}+c_{s}\right)\right)-\left(\frac{1}{2}{\left(a_{s}+c_{s}\right)}^{2}-{\left(a_{s}+c_{s}\right)}^{2}\right)\right)$. We can obtain $\frac{\partial^{2}\;v_{s}}{\partial \;a_{s}^{2}}=\beta -2<0$, which proves that $v_{s}$ is a concave function. Moreover, the constraint functions are affine. Thus, by employing the KKT condition, we can obtain the optimal price, as shown in Propositions 1 and 2.

**Proposition** **1.** *When the price ceiling $p_{0}$ satisfies $c_{s}\leq p_{0}\leq m_{s}$, the service price and total revenue are given, respectively, by (denoted by superscript $s_{1}$)*

(10) $$\begin{array}{cc} & p_{s}^{s_{1}}=p_{0}, \end{array}$$

(11) $$\begin{array}{cc} & v_{s}^{s_{1}}=p_{0}-p_{0}^{2}-c_{s}+c_{s}\cdot p_{0}+\beta \cdot \left(\frac{1}{2}-p_{0}+\frac{1}{2}\cdot p_{0}^{2}\right), \end{array}$$

*where $m_{s}=\frac{1+c_{s}}{2}$.*

**Proposition** **2.** *When the price ceiling $p_{0}$ satisfies $m_{s}\leq p_{0}\leq 1$, the optimal unit revenue, service price, and total revenue are given by (denoted by superscript $s_{2}$)*

(12) $$\begin{array}{cc} & a_{s}^{s_{2}}=\frac{\left(1-\beta \right)\cdot \left(1-c_{s}\right)}{2-\beta }, \end{array}$$

(13) $$\begin{array}{cc} & p_{s}^{s_{2}}=\frac{1+c_{s}-\beta }{2-\beta }, \end{array}$$

(14) $$\begin{array}{cc} & v_{s}^{s_{2}}=\frac{\left(1-\beta \right)\cdot {\left(1-c_{s}\right)}^{2}}{2-\beta }+\beta \cdot \left(\frac{1}{2}-\frac{1+c_{s}-\beta }{2-\beta }+\frac{1}{2}\cdot {\left(\frac{1+c_{s}-\beta }{2-\beta }\right)}^{2}\right). \end{array}$$

**Proof.**  Proof for Propositions 1 and 2.

The Lagrangian function of Equation (9) is

(15) $$\begin{array}{cc} \; & L\left(a_{s},c_{s},\lambda_{1},\lambda_{2}\right)=a_{s}\cdot q_{s}+\beta \cdot \int_{\delta_{s}}^{1}\left(\delta -p_{s}\right)d\delta +\lambda_{1}\left(p_{0}-p_{s}\right)+\lambda_{2}a_{s}\\ \; & =a_{s}\cdot \left(1-a_{s}-c_{s}\right)+\beta \cdot \left(\left(\frac{1}{2}-p_{s}\right)-\left(\frac{1}{2}p_{s}^{2}-p_{s}^{2}\right)\right)+\lambda_{1}\cdot \left(p_{0}-p_{s}\right)+\lambda_{2}\cdot a_{s}, \end{array}$$

where $\lambda_{1}$ and $\lambda_{2}$ denote the Lagrangian multipliers associated with the constraints in Equation (9). Through the KKT conditions, we have:

$$\begin{array}{cc} & \frac{\partial \;L(a_{s},c_{s},\lambda_{1},\lambda_{2})}{\partial \;a_{s}}=0,\\ & \frac{\partial \;L(a_{s},c_{s},\lambda_{1},\lambda_{2})}{\partial \;\lambda_{1}}\geq 0,\\ & \frac{\partial \;L(a_{s},c_{s},\lambda_{1},\lambda_{2})}{\partial \;\lambda_{2}}\geq 0,\\ & \lambda_{1}\cdot \left(p_{0}-p_{s}\right)=0,\\ & \lambda_{2}\cdot a_{s}=0,\\ & \lambda_{1},\lambda_{2}\geq 0. \end{array}$$

Obviously, $\lambda_{2}=0$; then, we discuss the case of Equation (12): When $\lambda_{1}=0$, we have $p_{s}<p_{0}$; let $\frac{\partial \;L(a_{s},c_{s},\lambda_{1},\lambda_{2})}{\partial \;a_{s}}=1-2\cdot a_{s}-c_{s}-\beta +a_{s}\cdot \beta +c_{s}\cdot \beta =0$, and we can obtain

$$a_{s}^{s_{2}}=\frac{\left(1-\beta \right)\cdot \left(1-c_{s}\right)}{2-\beta },$$

Thus, we can obtain $p_{s}^{s_{2}}=a_{s}^{s_{2}}+c_{s}=\frac{1+c_{s}-\beta }{2-\beta }$. When $\lambda_{1}>0$, obviously, we have $p_{0}=p_{s}$; thus, the single-channel service price has reached the price ceiling $p_{0}$. Then, we have

$$p_{s}^{s_{1}}=p_{0}.$$

Hence, the results are obtained. □

Proposition 1 shows that when $p_{0}$ is lower than $m_{s}$, the service price is independent of β. When $p_{0}$ is higher than $m_{s}$, channels are required to pay more attention to public welfare. Moreover, Proposition 2 shows that the service price closely depends on $p_{0}$ and β. In particular, the case of $p_{s}=m_{s}$ implies that the single channel is purely profit-oriented.

**Corollary** **1.** *For price ceiling $m_{s}\leq p_{0}\leq 1$, with the increase in β, $p_{s}^{s_{2}}$ and $v_{s}^{s_{2}}$ will reduce, while $CS_{s}^{s_{2}}$ will increase, and $\frac{CS_{s}^{s_{2}}}{v_{s}^{s_{2}}}>\frac{\pi_{s}^{s_{2}}}{v_{s}^{s_{2}}}$. For price ceiling $c_{s}\leq p_{0}<m_{s}$, β has no impact on the service price. $CS_{s}^{s_{2}}$ will reduce when increasing $p_{0}$, and $\frac{CS_{s}^{s_{1}}}{v_{s}^{s_{1}}}<\frac{\pi_{s}^{s_{2}}}{v_{s}^{s_{2}}}$.*

**Proof.**  When $m_{s}\leq p_{0}\leq 1$, $p_{s}^{s_{2}}=\frac{1+c_{s}-\beta }{2-\beta }$, we can easily obtain that $\frac{dp_{s}^{s_{2}}}{d\beta }=\frac{c_{s}-1}{{\left(2-\beta \right)}^{2}}<0$, $\frac{d\;CS^{s_{2}}}{d\;\beta }>0,\frac{d\;v_{s}^{s_{2}}}{d\;\beta }<0$. To simplify the presentation of the results, we define $\Delta_{1}=\frac{\pi_{s}^{s_{2}}}{v_{s}^{s_{2}}}$ and $\Delta_{2}=\frac{CS_{s}^{s_{2}}}{v_{s}^{s_{2}}}$ to represent the ratio of economic revenue and public welfare to single-channel total revenue separately. Let

$$F\left(\beta \right)=\Delta_{1}-\Delta_{2},$$

$$F(\beta_{1})=0,$$

and it is obvious that when $\beta <\beta_{1},$F(β)>0, when $\beta >\beta_{1},$F(β)<0, and we also have

$$\frac{d\;F(\beta )}{d\;\beta }<0$$

and

$$\frac{d^{2}\;F\left(\beta \right)}{d\;\beta^{2}}<0.$$

when $c_{s}<p_{0}\leq m_{s}$, $p_{s}^{s_{1}}=p_{0}$, we have $\frac{d\;CS^{s_{1}}}{d\;p_{0}}<0$. To simplify the presentation of the results, we define $\Delta_{3}=\frac{\pi_{s}^{s_{1}}}{v_{s}^{s_{1}}}$ and $\Delta_{4}=\frac{CS_{s}^{s_{1}}}{v_{s}^{s_{1}}}$ to represent the ratio of economic revenue and public welfare to single-channel total revenue separately. Let

$$T\left(p_{0}\right)=\Delta_{3}-\Delta_{4},$$

T(p)=0,

and it is obvious that when $p_{0}>p$, $F(p_{0})>0$, when $p_{0}<p$, $F(p_{0})<0,$ and we also obtain

$$\frac{d\;G(p_{0})}{d\;p_{0}}>0,$$

and

$$\frac{d^{2}\;G\left(p_{0}\right)}{d\;p_{0}^{2}}<0.$$

Hence, the results are obtained. □

Corollary 1 indicates that, compared with $p_{s}^{{}^{\prime }}$, channels’ public welfare is a benefit that allows patients to access healthcare services with a lower cost, and part of the channel’s economic revenue is transferred into patient surplus, thus reducing the channel’s total revenue. $CS_{s}^{s_{2}}$ takes a greater percentage in $v_{s}^{s_{2}}$ than $\pi_{s}^{s_{2}}$ when increasing β, which denotes that the increase in public welfare has gradually changed the healthcare institution to focus on public welfare. When $p_{s}=p_{0}$, channels’ economic revenue $\pi_{s}^{s_{1}}$ takes a greater percentage in $v_{s}^{s_{1}}$ with the loss of public welfare.

### 4.2. Decentralized Decision Scenario: Dual-Channel Strategy

In this subsection, we investigate the optimal strategy employed by the online and offline channels by considering the impact of the patients’ online channel acceptance, public welfare, and the government’s price ceiling. In this decentralized case, both channels pursue their maximum revenue. The results in this section are derived under the condition $p_{e}+1-\theta \rho >p_{r}>p_{e}$.

The healthcare institution, online channel, and offline channel constitute the dual-channel HSSC, in which the offline channel works as a Stackelberg game leader [34,35] due to the fact that the offline channel has more dominance in the market. Based on previous assumptions, the offline channel needs to solve the following decision optimization problem to seek its optimal price, shown as:

(16) $$\begin{array}{ccc} & \max & v_{r}^{d}=a_{r}\cdot q_{r}+\beta \cdot \int_{\delta_{d}}^{1}\left(\delta -p_{r}\right)d\delta ,\\ & s.t. & p_{e}<p_{r}\leq p_{0},\\ & & a_{r}\geq 0. \end{array}$$

Moreover, the online channel’s decision optimization problem is written as:

(17) $$\begin{array}{ccc} & \max & v_{e}^{d}=a_{e}\cdot q_{e}+\beta \cdot \int_{0}^{\delta_{d}}\left(\delta -p_{e}\right)d\delta ,\\ & s.t. & p_{e}\leq p_{r}\leq p_{0},\\ & & a_{e}\geq 0. \end{array}$$

It is worth mentioning that the total revenue of the dual channel depends on the optimal service price and public welfare coefficient in Equations (16) and (17). Constraints specify that the optimal service price should not exceed the price ceiling, and the unit revenue is non-negative. We employ backward induction [35] to solve the corresponding Stackelberg game between the online and offline channels. Specifically, we first solve the online channel decision problem in Equation (17) for given $a_{r}$ and find an optimal $a_{e}\left(a_{r}\right)$ formula for the leader’s decision; then, we solve the offline channel decision problem in Equation (16) for given $a_{e}\left(a_{r}\right)$ [36]. With particular attention to our problem, we use KKT [33,37] to find the optimal equilibrium solutions. For Equation (17), from $\frac{\partial^{2}\;v_{r}^{d}}{\partial \;a_{r}^{2}}=\frac{2(\beta -1)}{1-\theta \rho }-\frac{\beta }{1-\theta \rho }<0$, we obtain that $v_{r}^{d}$ is strictly concave in $a_{r}$. For Equation (16), from $\frac{\partial^{2}\;v_{e}^{d}}{\partial \;a_{e}^{2}}=\frac{2(\beta -1)}{1-\theta \rho }+\frac{\beta }{1-\theta \rho }$, we obtain that $v_{e}^{d}$ is also concave in $a_{e}$ if $\beta <\frac{3-2\theta \rho }{2-2\theta \rho }$. Therefore, when the optimality conditions are given to hold, the revenue of both the online and offline channels reaches the maximum. The decision maker takes the optimal decisions given in Proposition 3 below.

**Proposition** **3.** *When the service price satisfies $p_{e}<p_{r}<p_{0}$, the optimal service price (denoted by superscript $d_{1}$) can be obtained as follows:*

(18) $$\begin{array}{cc} \; & a_{e}^{d_{1}}=\frac{\left(\beta -1\right)\left(B-3t\right)c_{e}+\left(t-\beta (t+1)\right)\left(C+c_{r}\right)}{(1-\beta )\cdot B},\\ \; & a_{r}^{d_{1}}=\frac{-C^{2}+2\left(\beta -1\right)tc_{e}+\left(2t+D-6\beta t\right)c_{r}}{(\beta -1)\cdot B}, \end{array}$$

*where*

$$\begin{array}{cc} & t=1-\theta \rho ,\\ & B=4t-\beta (4t+1),\\ & C=2t-\beta (2t+1),\\ & D=\beta^{2}\left(1+4t\right). \end{array}$$

**Proposition** **4.** *When the service price satisfies $p_{r}=p_{0}$, we have $a_{r}^{d_{2}}=p_{0}-c_{r}$, and the online channel optimal price (denoted by superscript $d_{2}$) is given by*

(19) $$a_{e}^{d_{2}}=\frac{\beta \cdot c_{e}t-\left(\beta +(\beta -1)t\right)\left(p_{0}-c_{e}\right)}{C}.$$

**Proof.**  Proof for Propositions 3 and 4.

By setting the Equation (17) Lagrangian multipliers of the two constraints as $\varphi_{1}$ and $\varphi_{2}$, respectively, we can obtain the Lagrangian function:

$$\begin{array}{cc} \; & L(a_{e},a_{r},\varphi_{1},\varphi_{2})\\ \; & =a_{e}\cdot q_{e}+\beta \cdot \int_{0}^{\delta_{d}}\left(\delta -p_{e}\right)d\delta +\varphi_{1}\cdot \left(p_{0}-p_{e}\right)+\varphi_{2}\cdot a_{e}\\ \; & =\frac{a_{e}\cdot a_{r}+a_{e}\cdot c_{r}-a_{e}^{2}-a_{e}\cdot c_{e}}{1-\theta \rho }+\frac{1}{2}\beta \cdot \frac{a_{r}+c_{r}-a_{e}-c_{e}}{{\left(1-\theta \rho \right)}^{2}}-\beta \left(a_{e}+c_{e}\right)\cdot \frac{a_{r}+c_{r}-a_{e}-c_{e}}{1-\theta \rho }\\ \; & +\varphi_{1}\cdot \left(p_{0}-a_{e}-c_{e}\right)+\varphi_{2}\cdot a_{e}. \end{array}$$

The KKT conditions of Equation (17) are listed as follows:

$$\frac{\partial \;L(a_{e},a_{r},\varphi_{1},\varphi_{2})}{\partial \;a_{e}}\geq 0,$$

$$\frac{\partial \;L(a_{e},a_{r},\varphi_{1},\varphi_{2})}{\partial \;\varphi_{1}}\geq 0,$$

$$\frac{\partial \;L(a_{e},a_{r},\varphi_{1},\varphi_{2})}{\partial \;\varphi_{2}}\geq 0,$$

$$\varphi_{1}\cdot \left(p_{0}-p_{e}\right)=0,$$

$$\varphi_{2}\cdot a_{e}=0,$$

$$\varphi_{1},\varphi_{2}\geq 0.$$

Obviously, when $a_{e}=0$, there is no research significance; thus, we obtain $\varphi_{2}=0$. Moreover, through $p_{e}<p_{r}\leq p_{0}$, we can easily obtain $\varphi_{1}=0$. Then, the Lagrangian function of Equation (17) is written as:

$$\begin{array}{cc} & L\left(a_{e},a_{r},\varphi_{1},\varphi_{2}\right)=\frac{a_{e}\cdot a_{r}+a_{e}\cdot c_{r}-a_{e}^{2}-a_{e}\cdot c_{e}}{1-\theta \rho }+\frac{1}{2}\beta \cdot \frac{a_{r}+c_{r}-a_{e}-c_{e}}{{\left(1-\theta \rho \right)}^{2}}-\beta \left(a_{e}+c_{e}\right)\cdot \frac{a_{r}+c_{r}-a_{e}-c_{e}}{1-\theta \rho }. \end{array}$$

Let $\frac{\partial \;L(a_{e},a_{r},\varphi_{1},\varphi_{2})}{\partial \;a_{e}}=0$; then, the optimal solution is given by

(20) $$a_{e}=\frac{a_{r}\left(\beta (\theta \rho -2)-\theta \rho +1\right)+c_{e}\left(-2\beta \theta \rho +3\beta +\theta \rho -1\right)+c_{r}\left(\beta \theta \rho -2\beta -\theta \rho +1\right)}{\beta \cdot (2\theta \rho -3)-2\theta \rho +2}.$$

By setting the Equation (16) Lagrangian multipliers of the two constraints as $\gamma_{1}$ and $\gamma_{2}$, respectively, we can obtain $L(a_{r},a_{e},\gamma_{1},\gamma_{2})$. Then, we substitute Equation (20) into $L(a_{r},a_{e},\gamma_{1},\gamma_{2})$ and obtain:

$$\begin{array}{cc} \; & L(a_{r},\gamma_{1},\gamma_{2})\\ \; & =\gamma_{1}\left(p_{0}-a_{r}-c_{r}\right)+\gamma_{2}a_{r}-\frac{\left(-1+\beta \right)a_{r}\left(\left(-1+\beta \right)a_{r}+\left(-1+k+\beta \right)c_{r}\right)}{2-2\theta \rho +\beta (-3+2\theta \rho )}+\left(\frac{1}{2}-a_{r}-c_{r}\right)\beta \\ \; & \;-\frac{\beta c_{r}\left(\left(-1+\beta \right)a_{r}+\left(-1+k+\beta \right)c_{r}\right)}{2-2\theta \rho +\beta (-3+2\theta \rho )}-\frac{\beta {\left(\left(-1+\beta \right)a_{r}+\left(-1+k+\beta \right)c_{r}\right)}^{2})}{2{\left(2-2\theta \rho +\beta \left(-3+2\theta \rho \right)\right)}^{2}}+a_{r}. \end{array}$$

For $L(a_{r},\gamma_{1},\gamma_{2})$, via $\frac{\partial^{2}\;L\left(a_{r},\gamma_{1},\gamma_{2}\right)}{\partial \;a_{r}^{2}}=\frac{{\left(\beta -1\right)}^{2}\left(4-4\theta \rho +\beta (4\theta \rho -5)\right)}{{\left(2-2\theta \rho +\beta (2\theta \rho -3)\right)}^{2}}<0,$ we can obtain $\beta \in \left(0,\frac{4\theta \rho -4}{4\theta \rho -5}\right)\subseteq \left(0,\frac{2-2\theta \rho }{3-2\theta \rho }\right)$.

The KKT conditions of Equation (16) are:

$$\frac{\partial \;L(a_{r},\gamma_{1},\gamma_{2})}{\partial \;a_{r}}=0,$$

$$\frac{\partial \;L(a_{r},\gamma_{1},\gamma_{2})}{\partial \;\gamma_{1}}\geq 0,$$

$$\frac{\partial \;L(a_{r},\gamma_{1},\gamma_{2})}{\partial \;\gamma_{2}}\geq 0,$$

$$\gamma_{1}\cdot \left(p_{0}-p_{e}\right)=0,$$

$$\gamma_{2}\cdot a_{e}=0,$$

$$\gamma_{1},\gamma_{2}\geq 0.$$

Obviously, when $a_{r}=0$, it is meaningless; thus, we obtain $\gamma_{2}=0$. Then, we discuss the value of $\gamma_{1}$ separately.

**Case A.** When $\gamma_{1}=0$, we have $p_{r}<p_{0}$. By setting $\frac{\partial \;L(a_{r})}{\partial \;a_{r}}=0$, we can obtain

(21) $$a_{r}^{*}=\frac{{\left(\beta (2\theta \rho -3)-2\theta \rho +2\right)}^{2}+2\left(\beta -1\right)c_{e}\left(\theta \rho -1\right)-c_{r}\left(\beta^{2}\left(5-4\theta \rho \right)+6\beta \left(\theta \rho -1\right)-2\theta \rho +2\right)}{\left(1-\beta \right)\left(\beta (4\theta \rho -5)-4\theta \rho +4\right)}.$$

By substituting Equation (21) back into Equation (20), we can obtain

(22) $$\begin{array}{c} a_{e}^{*}=\frac{\left(\beta -1\right)c_{e}\left(\beta (4\theta \rho -5)-\theta \rho +1\right)+\left(\beta (\theta \rho -2)-\theta \rho +1\right)\left(\beta \left(2\theta \rho -3\right)+c_{r}-2\theta \rho +2\right)}{\left(1-\beta \right)\left(\beta (4\theta \rho -5)-4\theta \rho +4\right)}. \end{array}$$

To simplify the results, we denote t=1−θρ, B=4t−β(4t+1), C=2t−β(2t+1), $D=\beta^{2}\left(1+4t\right)$. Then, the optimal unit revenue of the online and offline channels when $p_{e}<p_{r}<p_{0}$ is given by:

(23) $$\begin{array}{cc} & a_{e}^{d_{1}}=\frac{\left(\beta -1\right)\left(B-3t\right)c_{e}+\left(t-\beta (t+1)\right)\left(C+c_{r}\right)}{(1-\beta )B}, \end{array}$$

(24) $$\begin{array}{cc} & a_{r}^{d_{1}}=\frac{C^{2}-2\left(\beta -1\right)tc_{e}-\left(2t+D-6\beta t\right)c_{r}}{(1-\beta )B}. \end{array}$$

By substituting Equations (21) and (22) into $p_{r}^{d_{1}}=a_{r}^{d_{1}}+c_{r},p_{e}^{d_{1}}=a_{e}^{d_{1}}+c_{e}$, we can obtain the optimal service price decision-making strategy. Then, the economic revenue and patient surplus are shown as follows:

$$\begin{array}{cc} & \pi_{e}^{d_{1}}=\frac{\left(C-c_{e}+c_{r}\right)\left(\left(\beta -1\right)\left(B-3t\right)c_{e}+\left(t-\beta (1+t)\right)\left(C+c_{r}\right)\right)}{\left(1-\beta \right)B^{2}},\\ & \pi_{r}^{d_{1}}=\frac{\left(2t\left(1-\beta \right)+c_{e}-c_{r}\right)\left(C^{2}+2tc_{e}\left(1-\beta \right)+\left(-2t+6\beta t-D\right)c_{r}\right)}{\left(1-\beta \right)B^{2}},\\ & CS_{e}^{d_{1}}=\frac{\left(C+c_{r}-c_{e}\right)\left(\left(1-\beta \right)\left(6t+1\right)c_{e}+\left(C-1\right)\left(C+c_{r}\right)\right)}{2\left(\beta -1\right)B^{2}},\\ & CS_{r}^{d_{1}}=\frac{\beta -\left(C-1\right)c_{r}^{2}+\left(1-\beta \right)\left(4t+1\right)c_{e}^{2}+4t\left(4t-1\right)c_{e}-2\beta c_{r}\left(\beta +2t+1\right)+2t\left(\left(-\beta +2\right)\beta -8{\left(\beta -1\right)}^{3}t^{2}+2\left(-\beta^{3}+\beta^{2}+\beta -3\right)t+2\right)}{2\left(\beta -1\right)B^{2}}. \end{array}$$

**Case B.** When $\gamma_{1}>0$, we have $p_{e}<p_{r}=p_{0}$. Obviously, we have $a_{r}^{d_{2}}=p_{0}-c_{r}$. By substituting $a_{r}^{d_{2}}=p_{0}-c_{r}$ into Equation (20), we can easily obtain:

(25) $$a_{e}^{d_{2}}=\frac{\beta c_{e}t-\left(\beta +(\beta -1)t\right)\left(p_{0}-c_{e}\right)}{C}.$$

By substituting Equation (25) into $p_{e}^{d_{2}}=a_{e}^{d_{2}}+c_{e}$, we can obtain the optimal service price decision-making strategy. Then, the economic revenue and patient surplus are shown as follows:

$$\begin{array}{cc} & \pi_{e}^{d_{2}}=\frac{\left(c_{e}+\left(\beta -1\right)p_{0}\right)\left(\left(C-t\right)c_{e}+\left(-t+\beta (t+1)\right)p_{0}\right)}{C^{2}},\\ & \pi_{r}^{d_{2}}=\frac{\left(p_{0}-c_{r}\right)\left(C+c_{e}+\left(\beta -1\right)p_{0}\right)}{C},\\ & CS_{e}^{d_{2}}=\frac{\left(c_{e}+\left(\beta -1\right)p_{0}\right)\left(\left(1+2t\right)c_{e}+\left(C-1\right)p_{0}\right)}{2C^{2}},\\ & CS_{r}^{d_{2}}=\frac{C^{2}-c_{e}^{2}-2C^{2}p_{0}-2\left(C+\beta -1\right)c_{e}p_{0}-\left(\beta -1\right)\left(B-1\right)p_{0}^{2}}{2C^{2}}. \end{array}$$

Hence, the results are obtained. □

**Corollary** **2.** *The increase in θ will cause the service price of the online and offline channel to decrease and then increase, and the growth of θ is more beneficial to the online channel.*

**Proof.**  For $a_{e}^{d_{1}}$ and $a_{r}^{d_{1}}$, we take the derivative of θ and make it 0, respectively, and then, we can obtain $\theta_{1}$ and $\theta_{2}$:

$$\frac{\partial \;a_{e}^{d_{1}}}{\partial \;\theta }=0,\theta =\theta_{1};$$

$$\frac{\partial \;a_{r}^{d_{1}}}{\partial \;\theta }=0,\theta =\theta_{2}.$$

when $\theta <\theta_{1},\theta <\theta_{2}$, $\frac{\partial \;a_{e}^{d_{1}}}{\partial \;\theta }<\frac{\partial \;a_{r}^{d_{1}}}{\partial \;\theta }<0$; when $\theta >\theta_{1},\theta >\theta_{2}$, $\frac{\partial \;a_{e}^{d_{1}}}{\partial \;\theta }>\frac{\partial \;a_{r}^{d_{1}}}{\partial \;\theta }>0$.

Hence, the results are obtained. □

Corollary 2 shows that the patients’ acceptance of the online channel θ has an impact on the service price of both the online and offline channels. With the increase in θ, the patients’ acceptance of the online channel gradually increases, and the demand for online services increases, causing the demand competition between the two channels to increase. The online channel needs a low-price strategy to attract patients, while the offline channel will decrease the service price to deal with the demand decrease, thus resulting in a decrease in both channels’ unit revenue. When θ increases to a higher level, the two channels achieve a relative demand balance. We can observe a win–win situation achieved between the two channels.

**Corollary** **3.** *When $p_{r}=p_{0}$, increasing the patients’ acceptance of the online channel can improve the patient surplus caused by patients visiting both channels, and it will also increase the channels’ total revenue. However, the economic revenue of both channels will decrease.*

**Proof.**  When $p_{r}=p_{0}$,

$$\frac{\partial \;CS_{e}^{d_{2}}}{\partial \;\theta }>0,\frac{\partial \;CS_{r}^{d_{2}}}{\partial \;\theta }>0,$$

$$\frac{\partial \;\pi_{e}^{d_{2}}}{\partial \;\theta }<0,\frac{\partial \;\pi_{r}^{d_{2}}}{\partial \;\theta }<0,$$

and for $v_{e}^{d_{2}}=\pi_{e}^{d_{2}}+CS_{e}^{d_{2}}$, $v_{r}^{d_{2}}=\pi_{r}^{d_{2}}+CS_{r}^{d_{2}}$, the partial derivatives of θ can be obtained: $\frac{\partial \;v_{e}^{d_{2}}}{\partial \;\theta }>0,$ $\frac{\partial \;v_{r}^{d_{2}}}{\partial \;\theta }>0.$

Hence, the results are obtained. □

Corollary 3 shows that increasing the patients’ acceptance of the online channel can lead to a win–win outcome for both providers and patients, with patients receiving more patient surplus and healthcare institutions receiving more total revenue, but with a decrease in financial gain for healthcare institutions.

It is clear that the service price decreases whenever the public welfare coefficient decreases. Thus, the patient can pay less for healthcare services. In the dual-channel strategy, the public welfare coefficient β has little impact on the economic revenue of both the online and offline channels. When the service price of the offline channel reaches the price ceiling, the economic revenue of the online channel will gradually decrease with the increase in β, and the motivation of healthcare institutions to open up online channels will also decrease. Moreover, the increase in the patients’ acceptance coefficient θ will increase both channels’ patient surplus. Thus, the increase in public welfare compensates for some of the decrease in economic revenue, but causes the economic revenue of the two channels to decrease slightly during the public welfare increase.

## 5. Numerical Analysis

To verify the influence of public welfare and patients’ channel acceptance coefficient on the optimal strategy, we begin by illustrating the strategy with the single channel using a numerical example and then discuss the dual-channel strategy with the online channel and offline channel. Consider the healthcare channel with the following parameters for cost and patients’ recovery degree through the online channel: $c_{s}=0.1$, $c_{r}=0.1$, $c_{e}=0.08$, and ρ=0.8. The other system parameters are provided in each numerical figure and table accordingly. It is noted that all the set parameter values are so chosen such that the concavity criteria in Section 4.2 and the assumptions remain valid for all the game models.

### 5.1. Analysis of Different Channel Strategies

In this subsection, we compare the service price and the total revenue of the HSSC that the healthcare institution can obtain in different channel strategies with different public welfare coefficients β and different channel acceptance θ, as shown in Table 3, Table 4, Table 5 and Table 6. From Table 3 and Table 4, we can observe that for any β, the service price in the single-channel strategy is significantly higher than that in the dual-channel strategy, e.g., in the case of β=0.3, the service price of the single channel is 0.47, while the dual channel’s average service price is 0.20. This is due to the competition between the channels’ demands brought by patients’ channel preferences. This also agrees with our expectation that patients can obtain more healthcare revenue under the dual-channel HSSC strategy. Further, it is worth noting that the revenue of the dual-channel HSSC is equal to or higher than that of the single-channel HSSC, which implies that the dual-channel strategy is beneficial to the healthcare industry, and healthcare institutions should be encouraged to open up online channels.

From Table 5 and Table 6, we can see that, for any θ, the service price in the single-channel strategy is also significantly higher than that in the dual-channel strategy, e.g., in the case of θ=0.6, the service of the single channel is 0.47, while the dual channel’s average service price is 0.25. We can still attribute this to the competition between channels. Moreover, this competition increases as θ increases, which arises from the patients’ gradual acceptance of the online channel. Then, the original monopoly pattern is broken by the competition, making low-cost healthcare services more accessible. Additionally, with the decrease in the service price, the total revenue of either the online or the offline channel declines and is lower than the total revenue of the single channel. We can explain this from two aspects: one is that the price of the single-channel strategy is much higher, which leads to excessive economic returns and, thus, increases the total income; the other is that the channel competition under the dual-channel strategy will lead to profit losses for the healthcare institution. To maintain the stability of the healthcare industry in the development process, the government should subsidize the loss of channel income caused by the opening of online channels. Thus, we can conclude that the development of the healthcare industry needs the guidance and financial support of the government.

### 5.2. The Impact of the Public Welfare Coefficient β on the Optimal Service Price

**Figure 4:**
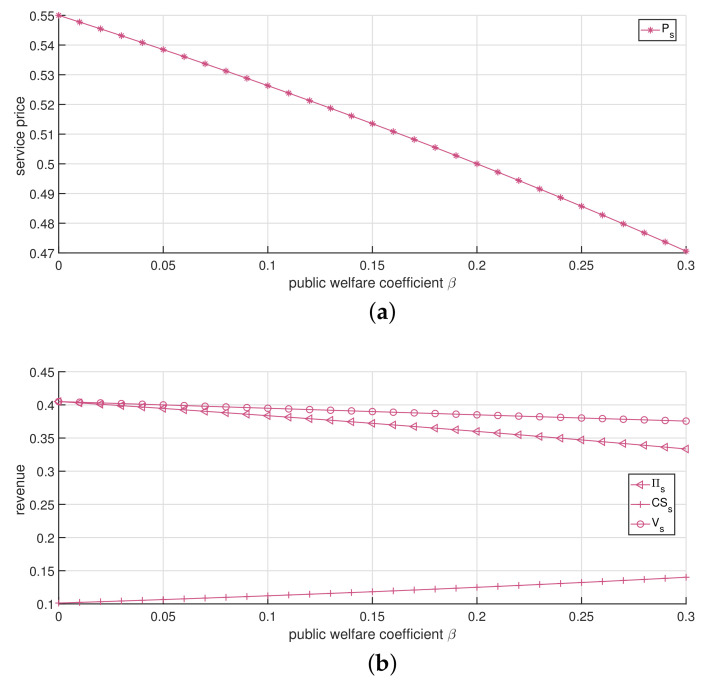
The impact of changes in β by employing the single-channel strategy. (**a**) The impact of changes in β against service price. (**b**) The impact of changes in β against revenue.

In this subsection, we analyze the impact of the public welfare coefficient β on the optimal service price and channels’ total revenue. We consider θ=0.7, which means that patients maintain a relatively high acceptance level for the online channel. Figure 4 depicts the optimal solution under the single-channel strategy. It shows in Figure 4b that as β increases, the patients’ surplus increases, whereas the single-channel’s economic revenue and total revenue decrease. Moreover, the rate of decline in total revenue is lower than that in economic revenue. The net result is that patients receive more benefits due to the increase in healthcare institutions’ public welfare, which also agrees with the expectation that the healthcare institution can improve patients’ satisfaction by taking more social responsibility. Furthermore, healthcare institutions will give up part of the economic revenue to ensure the public welfare of healthcare services, thus causing a decline in total revenue.

**Figure 5:**
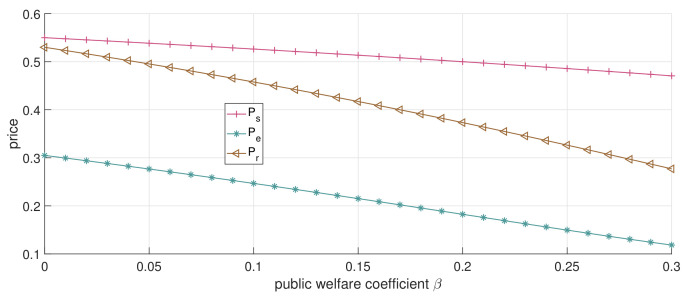
The impact of changes in β on service price (dual-channel strategy $p_{0}=0.8$).

**Figure 6:**
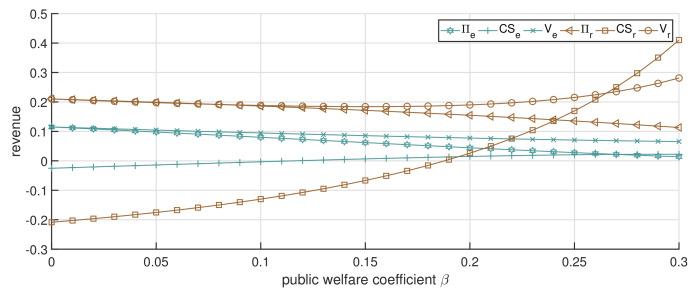
The impact of changes in β on CS and *V* (dual-channel strategy $p_{0}=0.8$).

When the dual-channel strategy is adopted and $p_{0}=0.8$, as Figure 5 and Figure 6 illustrate, a similar trend is observed with the increase in β compared to Figure 4a. The service price of both the online and offline strategies decreases, while the patients’ surplus increases. We can confirm again that the service price under the dual-channel strategy is lower than that of the single-channel strategy, and with the increase in β, the service prices of the offline channel and online channel ($p_{r}$ and $p_{e}$) decline faster than the single-channel service price ($p_{s}$). This shows that, under the dual-channel strategy, the increase in the public welfare coefficient is more conducive to patients’ access to low-cost healthcare services.

It is worth noting that the economic revenue of both the online and offline channels is trending downward, as illustrated in Figure 6, but the total revenue is different. Specifically, when β<0.15, with the increment in β, the total revenue of both the online and offline channels shows a slightly decreasing trend; when β>0.15, with the increment in β, the total revenue of the online channel still decreases, but the total revenue of the offline channel distinctly increases. This is mainly due to the patient surplus growth rate of patients in the offline channel being significantly greater than that in the online channel, resulting in the decline in the economic revenue of the offline channel. We can also observe from the curve trend of patients’ surplus that the influence of β on the offline channel is greater than that of the online channel. This implies that when the public welfare coefficient increases by the same amount, the public welfare revenue generated by the offline channel will be greater. However, when β is relatively low, the patient surplus obtained from the online channel is greater than that of the offline channel. It is the high price of services in the offline channel that leads to a low surplus for patients visiting. This means that the offline channel can take greater social responsibility by enhancing the coefficient of public welfare. From another point of view, the patient surplus in the online channel is not greatly affected by the increase in β, which means that, as the follower in the dual-channel game, the online channel will not focus on social responsibility, but on the replenishment of the offline channel. Additionally, this also confirms the national guidance on the positioning of the “Internet+” hospital.

**Figure 7:**
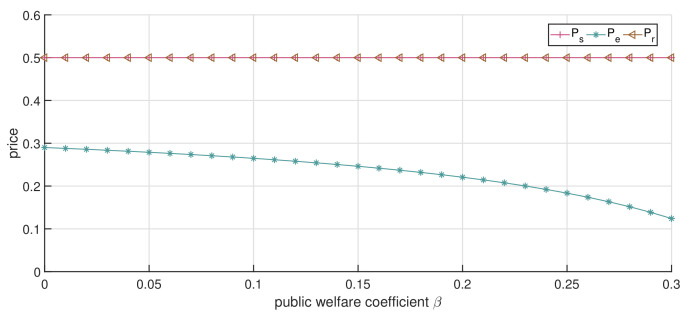
The impact of changes in β on service price (the dual-channel strategy $p_{0}=0.5$).

**Figure 8:**
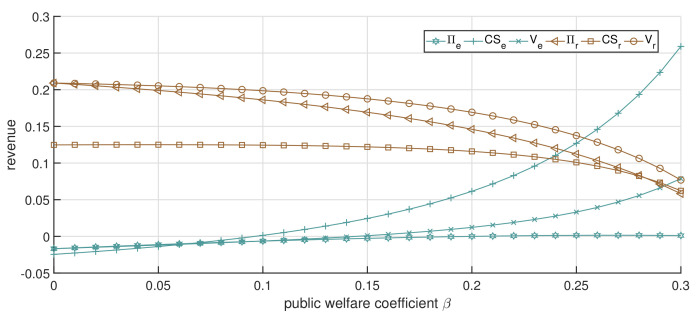
The impact of changes in β on CS and *V* (the dual-channel strategy $p_{0}=0.5$).

Figure 7 shows how the optimal price is affected by β when the price ceiling $p_{0}=0.5$. As shown in Figure 7, with the increase in β, the online channel’s service price decreases, which is similar to the results in Figure 5, while the offline channel’s service price reaches the price ceiling. This is because the offline channel is in the dominant position in the game model, and the institution tries to increase service prices as much as possible during the game process, while the online channel is in a subordinate position in the game model and then is affected by the price ceiling slightly. Figure 8 shows that with the increase in β, the total revenue of the offline channel decreases, whereas that of the online channel increases. In particular, when β is small, e.g., β<0.2, the total revenue of the offline channel is greater than that of the online channel. However, when β increases to a certain value, the total revenue of the online channel will be greater than the total revenue of the offline channel. This is because, with the increase in public welfare β, the service price of the online channel gradually decreases, while that of the offline channel remains unchanged. As such, patients gradually choose the online channel after comparing the acceptance of the online channel and service price, increasing the demand for the online channel. This has also led to an increase in the economic revenue of the online channel in the healthcare services market. Moreover, the low-priced service increases the patient surplus and the public welfare of the channel, which is reflected in the upward trend of the online channel’s total revenue, while the offline channel’s total revenue shows a downward trend. Thus, we can conclude that the substitutability of the offline channel is strong after considering the given acceptance of the online channel. In other words, when patients have a high degree of acceptance of the online channel, the offline channel will be in an unfavorable market position when the service price reaches the price ceiling. Exorbitant service prices cause patients to turn to the online channel to seek alternative healthcare services, realizing the diversion effect of dual-channel healthcare services supply.

### 5.3. The Impact of the Channel Acceptance θ on the Optimal Service Price

In this subsection, we analyze the impact of channel acceptance θ on the optimal service price and the channel’s total revenue. We consider β=0.3, which means that the public welfare coefficient is set at a high public welfare level.

**Figure 9:**
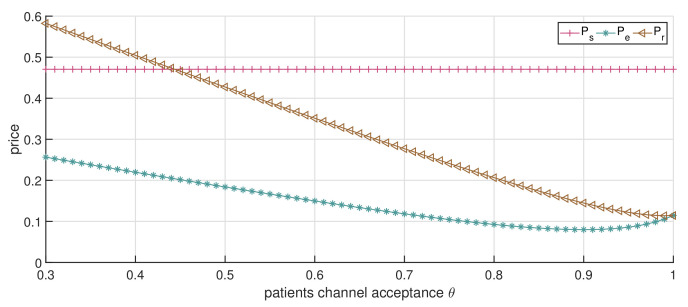
The impact of changes in θ on service price (dual-channel strategy $p_{0}=0.8$).

Figure 9 shows how the service price changes with the increase in θ when the price ceiling $p_{0}=0.8$. With the channel acceptance θ increasing, the online channel’s service price first decreases and then increases. The offline channel’s service price first decreases and then remains the same. It is noted that the service price of the online channel starts to increase when θ=0.9, and the service price of the online channel and the offline channel is similar when θ=1. These phenomena reflect changes in competition between channels as θ increases.

More specifically, when patients’ acceptance of the online channel is poor (e.g., θ∈[0.3,0.6]), the offline channel has the absolute advantage in the dual-channel supply, as the online channel strives for patient volume when the competition between the dual channels increases. In this case, the online channel has to decrease its service price to compete with the online channel, since fewer patients are choosing the online channel. Consistent with the non-service product market, patients are attracted to the lower price and choose online channel services. With the increment in θ, e.g., θ∈(0.6,0.8], patients gradually accept the online channel for healthcare treatment, causing the intensified demand competition between the online and the offline channel. This competition in turn results in a further reduction in service price, which is also consistent with the theory of market competition. As the patients’ acceptance of the online channel further increases, e.g., θ∈(0.8,1], the difference in their perceptions of the two channels becomes smaller. The number of patients choosing online channels has increased, followed by an increase in the service price, as the patients’ acceptance of online channels is close to 1, which means that the patients’ perceptions of different channels are almost the same. Thus, in this case, patients will choose channels for healthcare services according to their own needs, which alleviates the phenomenon of channel congestion and provides support for patient shunting. This also implies that the promotion and popularization of online channels are conducive to the efficient use of healthcare resources. This encourages the healthcare institution and the government to enhance their publicity and improve patients’ acceptance of online channels.

**Figure 10:**
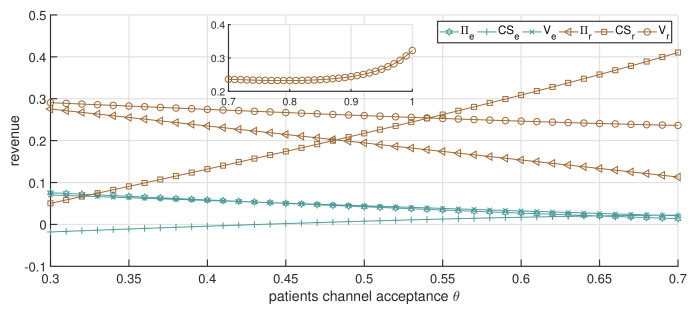
The impact of changes in θ on CS and *V* (dual-channel strategy $p_{0}=0.8$).

As illustrated in Figure 10, the total revenue of the online channel declines, while the total revenue of the offline channel shows a trend of first decreasing slightly and then increasing. These declines are caused by the decrease in channels’ economic revenue, which arises from the competition between channels, while the increases in public welfare cause the increases. The public welfare revenue of both the online and offline channels increases with the increase in θ, while the increase of the offline channels occurs much faster. This is mainly due to the rehabilitation effect of the offline channel being better than that of the online channel. The drop in the service price of the offline channel will bring more patient surplus to patients, and the online channel’s service price decreases lead to a slight increase. This implies an increase in patient acceptance of online channels, lowering the price and increasing the accessibility of healthcare services. Patients choose their access channel for greater patient surplus. Objectively, this also promotes the further development of both the online and offline channels.

**Figure 11:**
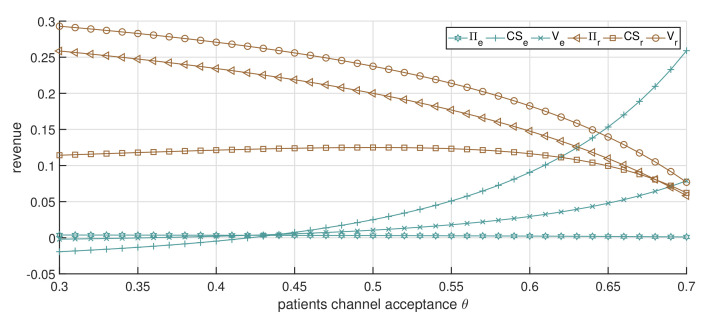
The impact of changes in θ on CS and *V* (the dual-channel strategy $p_{0}=0.5$).

As illustrated in Figure 11, when the offline channel’s service price reaches $p_{0}$, the total revenue of the online channel increases while that of the offline channel decreases with the increase in θ. This is mainly because their public welfare changes differently. We can see that the online channel’s public welfare $CS_{e}$ increases with the increase in θ, while the offline channel’s public welfare $CS_{r}$ slightly increases when θ∈(0,0.55) and then decreases when θ∈(0.55,0.7). This is because, when θ is low, offline channels are still the first choice for patients when seeking healthcare treatment. However, with the increase in θ, patients are willing to adopt online channels, and $p_{e}<p_{0}$; they can obtain low-cost, homogeneous healthcare services, resulting in a gradual increase in the patients’ surplus in online channels.

## 6. Conclusions

In this paper, the price decision-making problem of a dual-channel HSSC was taken as the research object. The optimal price decision-making strategy was considered when patients’ acceptance of online channels and the public welfare coefficient change, as well as the channel’s total revenue from the healthcare service. Firstly, we constructed the price decision-making model for healthcare institutions with only offline channels as a benchmark. Then, after the introduction of the online channel, we established a framework to study the strategic roles of the service price in the dual-channel HSSC and observed the effect on their revenue and patients’ surplus. According to Stackelberg game theory, we proposed different optimal price decision-making strategies considering the cases subject to the price ceiling constraint and not subject to the price ceiling constraint, respectively. Finally, we used a numerical example to verify the influence of the change in the public welfare coefficient and patients’ acceptance on the optimal price decision-making strategy and revenue of each channel under the different strategies. Above all, we obtained the following theoretical results and managerial implications from the perspective of public welfare and the sustainable promotion of a dual-channel HSSC:

(1) Compared with the single-channel strategy for healthcare services, the application of the dual-channel strategy can decrease the healthcare service price, enrich patients’ access options, and increase patient surplus. By comparing the optimal price decision-making strategy under the single-channel and dual-channel strategy, we found that the healthcare service price under the dual-channel strategy is smaller than that under the single-channel strategy. On the other hand, although adopting the dual-channel strategy slightly reduced the economic revenue of the healthcare institution, it achieved a significant increase in social welfare, which is the essence and social responsibility of the healthcare industry. It also reflects the sustainable development of healthcare service supply through the increase in social responsibility.
(2) We found that the public welfare coefficient strongly influenced the healthcare service price and channel revenue (including public welfare and economic revenue). When the price of the offline channel’s services did not reach the price ceiling, increasing the public welfare coefficient can decrease the price and increase social welfare, which is beneficial to patients. However, the government and healthcare institutions should also reasonably determine the public welfare coefficient to control the amount of economic losses of different channels. When the offline channel’s service price reaches the price ceiling, the increase in the public welfare coefficient decreases the online channel’s service price. Nevertheless, the offline channel’s service price remains unchanged. With the homogeneity of services, the online channel’s low-priced services affect the service demand of offline channels, which leads to a decrease in offline channels’ revenue.
(3) The acceptance of online channels also affects the optimal decision of the dual-channel strategy also, which is an important factor considered in constructing the healthcare services online channel and sustainable development. We noticed that the increased acceptance of online channels was conducive to improving the dual-channel HSSC and promoting healthy competition between channels. For example, when the offline channel’s service price did not reach the price ceiling, with the increase in patients’ acceptance of online channels, both channels needed to decrease their service price simultaneously to compete for their channel demand. When the acceptance of the online channel was in proximity to the offline channel, both channels’ service price rebounded. This indicates that an improvement in the supply method and the popularization of the online channel can help patients obtain sufficient patient surplus. In contrast, when the offline channel’s service price reaches the price ceiling, the service price decreases as the patients’ acceptance of the online channel increases. When the offline channel’s service price reaches the price ceiling, this places the offline channel at a disadvantage in the healthcare services market; thus, the price ceiling setting should be carefully chosen. Improving patient acceptance of online channels will not only help patients to gain access to more service channels and low-cost healthcare services, but also alleviate channel congestion, optimize resource allocation, and assume more social responsibility for healthcare institutions, which is beneficial for the sustainable development of healthcare service supply.
(4) Our research conclusions on the dual-channel HSSC provide theoretical support for the price decision-making strategy of healthcare institutions and relevant government departments. The results of this paper can help healthcare institutions and government departments balance economic revenue and public welfare revenue in the setting of dual-channel healthcare service prices, efficiently allocate medical resources, and improve patients’ satisfaction with healthcare services.

Aiming at the service price decision-making optimization of a dual-channel HSSC, we provided an optimization model for the online and offline channels. We analyzed the impacts of the public welfare coefficient and patients’ acceptance of the online channel on channels’ pricing decisions, revenue, and public welfare. With the more strategic allocation of healthcare resources, healthcare services will be more accessible to patients at affordable prices, which benefits the sustainability of the healthcare supply. In this paper, we considered the same public welfare coefficient only. Moreover, we did not consider health insurance policies in the proposed dual-channel structure and the objective function, which need to be further expanded by scholars in the future. Firstly, health insurance policies can be introduced to make the model closer to reality, with practical significance. Secondly, the channel acceptance of patients can be expanded to consider the situation wherein patients prefer to accept online channels rather than offline channels. In terms of the research method, this paper did not analyze the game model dominated by online medical service channels. Follow-up research should focus on this case, where online channels are dominant. The optimization method adopted in this paper requires the optimization model to be a convex optimization model, and the follow-up research should also consider the solution and analysis of the non-convex optimization model. Furthermore, in presenting the research conclusions, this paper lacks an analysis of the simultaneous changes in multiple factors. Subsequent research will focus on analyzing the impact of decision-making under simultaneous changes in multiple factors. Hence, it would be interesting to investigate the allocation scenario under the dual-channel strategy. Finally, the dual-channel HSSC model can be improved by considering the uncertainty of demand for the patient group on each channel to address the influence on decision-making optimization in the future.

## Figures and Tables

**Table 1:** Symbols and notations used in the paper.

Parameters	Descriptions
$q_{e}$	Patients’ demand for online healthcare services
$q_{r}$	Patients’ demand for offline healthcare services
μ	Initial utility when the patient is healthy
θ	Patients’ acceptance of online channel, within the range of (0,1)
β	Healthcare services’ public welfare coefficient
δ	The severity of the patients’ illness, evenly distributed in the range of [0,1)
ρ	The rehabilitation level of patients after receiving healthcare services through online channels, within the range of (0,1)
$p_{e}$	Price of online channel healthcare services
$p_{r}$	Price of offline channel healthcare services
$p_{0}$	Government’s price ceiling for healthcare services
$U_{e}$	Utility from online healthcare services channel
$U_{r}$	Utility from offline healthcare services channel
$\pi_{e}$	Economic revenue of online healthcare services
$\pi_{r}$	Economic revenue of offline healthcare services
$CS_{e}$	Online channel patient surplus
$CS_{r}$	Offline channel patient surplus
$v_{e}$	Total revenue of the online channel
$v_{r}$	Total revenue of the offline channel
$c_{e}$	Unit cost of online healthcare services channel
$c_{r}$	Unit cost of offline healthcare services channel

**Table 2:** Research methods used in this paper.

Scenario	Research Methods
Benchmark Scenario: Single-channel strategy	(1). KKT optimality
Decentralized Decision Scenario: Dual-channel strategy	(1). Stackelberg game—offline channel dominating (2). Backward induction (3). KKT optimality

**Table 3:** Performance comparison under different channel strategies (θ=0.7, $p_{0}=0.8$).

Item	$p_{s}$	$p_{e}$	$p_{r}$	$V_{s}$	$V_{e}$	$V_{r}$
β=0.25	0.49	0.15	0.33	0.38	0.07	0.22
β=0.3	0.47	0.12	0.28	0.38	0.07	0.28
β=0.4	0.44	0.08	0.18	0.37	0.06	0.88

**Table 4:** Performance comparison under different channel strategies (θ=0.6, $p_{0}=0.8$).

Item	$p_{s}$	$p_{e}$	$p_{r}$	$V_{s}$	$V_{e}$	$V_{r}$
β=0.25	0.49	0.19	0.40	0.38	0.05	0.21
β=0.3	0.47	0.15	0.35	0.38	0.03	0.25
β=0.4	0.44	0.09	0.24	0.37	0.02	0.67

**Table 5:** Performance comparison under different channel strategies (β=0.3, $p_{0}=0.8$).

Item	$p_{s}$	$p_{e}$	$p_{r}$	$V_{s}$	$V_{e}$	$V_{r}$
θ=0.5	0.47	0.18	0.43	0.38	0.04	0.26
θ=0.6	0.47	0.15	0.35	0.38	0.03	0.25
θ=0.7	0.47	0.12	0.28	0.38	0.02	0.24

**Table 6:** Performance comparison under different channel strategies (β=0.25, $p_{0}=0.8$).

Item	$p_{s}$	$p_{e}$	$p_{r}$	$V_{s}$	$V_{e}$	$V_{r}$
θ=0.5	0.49	0.22	0.48	0.38	0.06	0.23
θ=0.6	0.49	0.19	0.40	0.38	0.05	0.21
θ=0.7	0.49	0.15	0.33	0.38	0.03	0.18

## Data Availability

Not applicable.
